# Burn Pits: A Possible Trigger for Achalasia

**DOI:** 10.7759/cureus.36071

**Published:** 2023-03-13

**Authors:** Paul M Travers, Dawn L Francis

**Affiliations:** 1 Department of Internal Medicine, Mayo Clinic, Jacksonville, USA; 2 Division of Gastroenterology and Hepatology, Mayo Clinic, Jacksonville, USA

**Keywords:** military, esophageal manometry, esophageal dysmotility, regurgitation, gastroenterology, achalasia

## Abstract

A 45-year-old female veteran of the United States Air Force (USAF), who was exposed to burn pits on multiple occasions while deployed in the Middle East, presented for a second opinion regarding ongoing chest pain and regurgitation after a Heller myotomy for achalasia.

An esophageal X-ray showed no meaningful peristalsis, a slight diverticulum in the distal esophagus, and easy passage of liquids through the lower esophageal sphincter (LES). Esophageal manometry findings were consistent with type 3 achalasia.

Based on these and endoscopic evaluation, the prior surgical intervention appeared to be successful for lower esophageal sphincter disruption, so symptoms were managed medically with a proton pump inhibitor, trazodone, and a long-acting nitrate resulting in 70% improvement.

We present this case because the patient developed achalasia with a notable history of exposure to open-air burn pits during her military service. While we acknowledge that causality cannot be proven, our case is the first we are aware of that shows a temporal association between burn pit exposure and achalasia. In August of 2022, the United States Congress passed the Promise to Address Comprehensive Toxics (PACT) Act, which expanded the healthcare benefits of veterans exposed to burn pits, making identification of associated conditions a relevant and important endeavor.

## Introduction

Primary achalasia is a disease of esophageal dysmotility characterized by aperistalsis and impairment of lower esophageal sphincter (LES) relaxation secondary to the degradation of neurons in the myenteric plexus [1]. Although well studied, the underlying pathophysiologic mechanisms are incompletely understood.

The current leading theory is that insults to the esophageal mucosa (viral infections, parasitic infections, and environmental exposures) lead to localized inflammation of the myenteric plexus, causing the activation of an autoimmune cascade in genetically susceptible individuals [2]. This cascade then leads to the degeneration of primarily nitrous oxide (NO) and vasoactive intestinal peptide (VIP) producing inhibitory neurons [3,4]. Although this theory is becoming widely accepted, the distinct environmental exposures and/or genetic components that lead to the selective destruction of the inhibitory neurons of the myenteric plexus have yet to be comprehensively identified [5].

We present the case of a patient diagnosed with achalasia with a notable history of prolonged exposure to burn pits during military service. The Promise to Address Comprehensive Toxics (PACT) Act is an act signed into law in August of 2022 intended to improve healthcare coverage for veterans exposed to toxic substances during their time of service, including burn pits and Agent Orange. Exposure to these toxic chemicals has been linked to numerous medical conditions, including respiratory and cardiovascular diseases, as well as certain types of cancer. Further research is needed to better understand the potential link between burn pit exposure and achalasia, as well as other medical conditions.

## Case presentation

A 45-year-old female veteran of the United States Air Force (USAF) who had exposure to burn pits over a 20-year period of service presented for a second opinion regarding previously diagnosed achalasia. The patient’s symptoms began at the age of 26, eight years into her military service, during a time when she was deployed within 10 miles of a burn pit. Her early symptoms included acid reflux, chest tightness, and dysphagia to solid food. She was then relocated to an air force base on US soil, during which time her symptoms improved. During her last deployment (15 years into her career), she was living within five miles of a large burn pit with daily exposures in close proximity. It was during this deployment that her symptoms rapidly progressed to the point of dysphagia in both solids and liquids. These symptoms ultimately lead to her retirement from the USAF. Of note, she endorsed a family history of two maternal aunts with achalasia, although only one aunt had completed diagnostic testing with esophageal manometry. There was no known family history of Allgrove syndrome or Alport syndrome.

Previous evaluation for the patient’s symptoms had included otolaryngology evaluation with a diagnosis of vocal cord dysfunction, a diagnosis of asthma and then refuting of the diagnosis of asthma, a diagnosis of reflux and then refuting of the diagnosis of reflux, and finally evaluation by a surgeon who diagnosed her with achalasia via high-resolution esophageal manometry (HRM) and completed laparoscopic Heller myotomy with Dor fundoplication. She was 44 years old at the time. Upon evaluation at our center six months post-surgery, she continued to experience debilitating dysphagia, reflux, and chest tightness.

**Figure 1:**
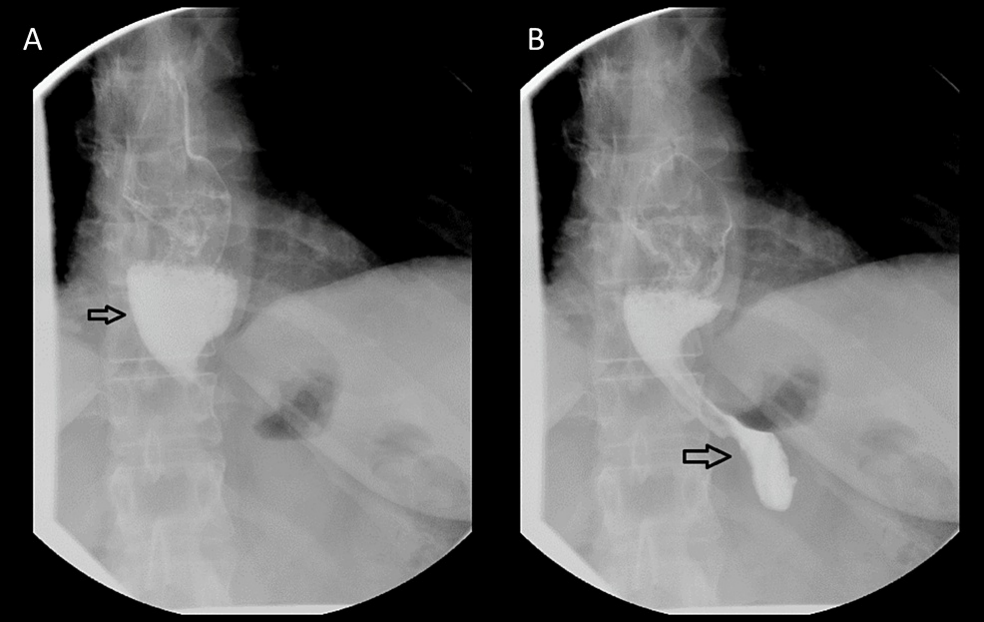
Barium Esophagram Single-contrast barium esophagography showing (A) no meaningful esophageal peristalsis consistent with a diagnosis of achalasia and (B) a slight diverticulum in the distal esophagus, which did not inhibit passage of liquids through the Heller myotomy/fundoplication

**Figure 2:**
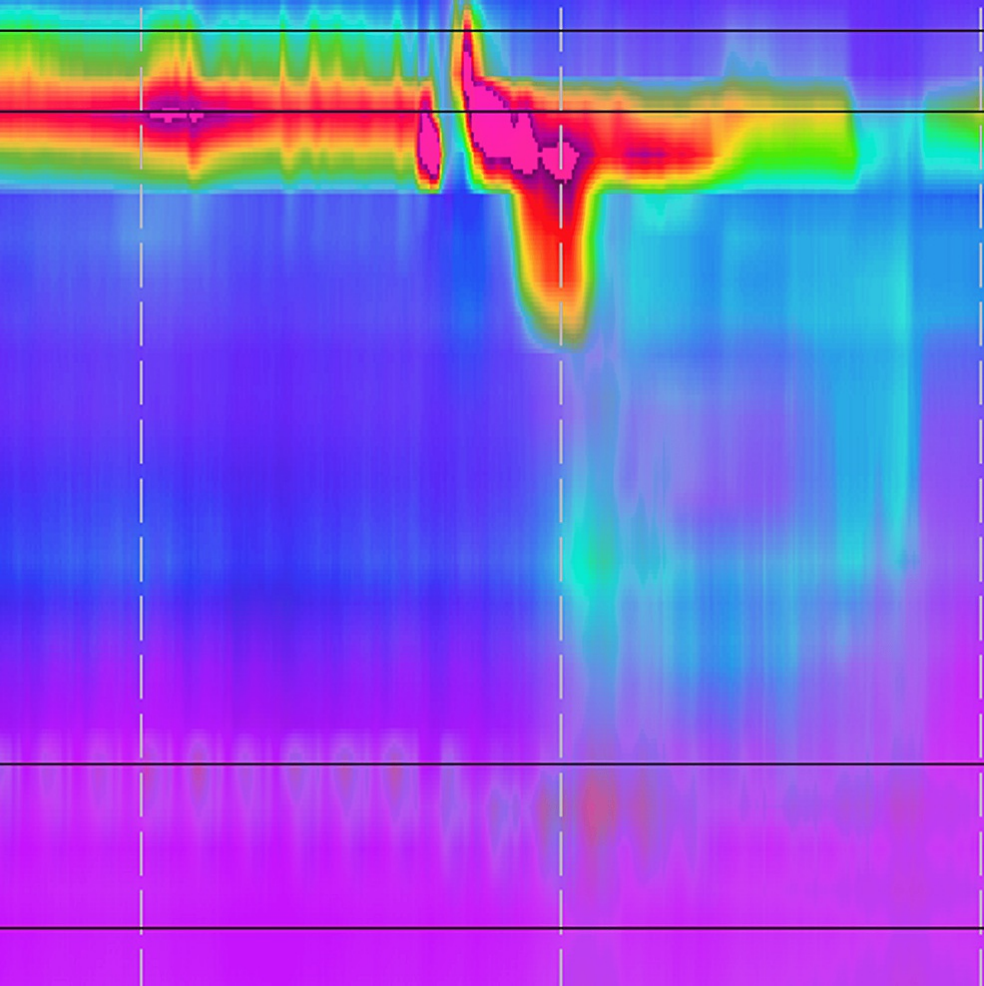
Esophageal Manometry High-resolution esophageal manometry showing no meaningful peristalsis, consistent with a diagnosis of achalasia

**Figure 3:**
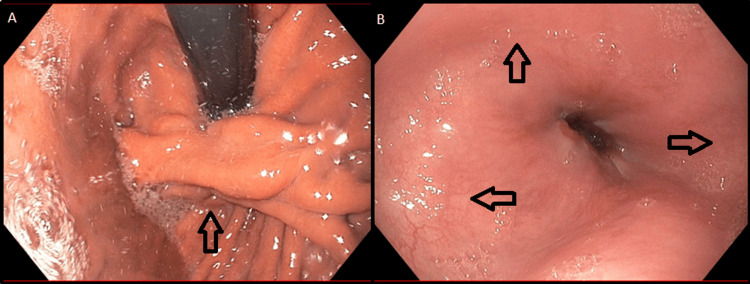
Esophagogastroduodenoscopy Esophagogastroduodenoscopy showing (A) evidence of Heller myotomy with Dor fundoplication wrap appearing below the gastroesophageal junction and (B) mild dilation of the lower third of the esophagus

As a part of our initial evaluation, we completed a number of investigations. A barium esophagram showed no meaningful peristalsis and a slight diverticulum in the distal esophagus, which did not inhibit the passage of liquids through the lower esophageal sphincter (LES) (Figure 1). Esophageal manometry showed a median integrated relaxation pressure (IRP) of 6.8 mmHg and 30% premature contractions with no meaningful peristalsis but a number of isobaric contractions consistent with a diagnosis of achalasia (Figure 2). Of note, this manometric testing was completed after Heller myotomy and Dor fundoplication. Esophagogastroduodenoscopy (EGD) was notable for fundoplication wrap appearing below the gastroesophageal junction with identification of exposed suture of eroded Ethibond (Figure 3). The lower esophageal sphincter was seen to be open. The patient was offered a revisional operation for slipped Dor fundoplication but opted for continued medical management.

The patient was initially started on omeprazole 40 mg two times daily in an attempt to limit symptoms of regurgitation. She completed eight weeks of this therapy without improvement of symptoms. She had completed a gastric emptying study at an outside institution and had reportedly been diagnosed with gastroparesis in the interim. Omeprazole was discontinued, and trazodone 50 mg nightly was started with the thought that some of her symptoms may be due to retropulsion or spasms. On follow-up six weeks later, the patient reported a mild improvement in feelings of chest tightness but worsening of reflux, so the dose of trazodone was increased to 50 mg twice daily and omeprazole was restarted. This suggested that antispasmodics may be helpful, so sublingual nitroglycerin before meals was also started.

She maintained moderate symptom control on this regimen for approximately six months. She then returned and reported that the combination of omeprazole, trazodone, and as-needed nitroglycerin was no longer controlling her symptoms and that she was experiencing headaches with each dose of nitroglycerin. She was therefore continued on trazodone and switched to tadalafil 5 mg every other day. This did not improve her symptoms, so the tadalafil was discontinued, and she was started on isosorbide mononitrate 30 mg daily. On follow-up one month later, she reported significant improvement in her symptoms of chest tightness, with a 90% decrease in the frequency of episodes.

This combination kept her symptoms under control for another six months. When she returned, she reported significant worsening of dysphagia and regurgitation now with additional episodes of gurgling and food impaction resolved with vomiting. A repeat barium esophagram was obtained, which showed a dilated esophagus with no effective primary peristalsis. She underwent an EGD, which showed a lack of normal peristalsis in the lower third of the esophagus. One hundred units of botulinum toxin were injected into the LES, and the area was dilated to 20 mm.

## Discussion

Achalasia is a rare but well-studied disorder of esophageal dysmotility, occurring equally in males and females with a predicted incidence of one in 100,000 [6]. The common presenting symptoms include dysphagia, regurgitation, weight loss, chest pain, heartburn, nocturnal cough, and aspiration, with dysphagia being the most prevalent [6]. The underlying pathophysiology involves the loss of a coordinated peristaltic wave and relaxation of the lower esophageal sphincter (LES) secondary to selective degeneration of inhibitory neurons of the myenteric plexus, specifically, the ganglionic cells of the esophageal body and LES [2,7]. Numerous studies have shown that the destruction of these cells is accompanied by a localized inflammatory response, leading to the theory that achalasia develops following mucosal disruption secondary to environmental exposure such as infection, autoimmune reaction, or chronic mucosal injury [2,8,9]. There seems to be a genetic component, although achalasia does not seem likely to be an exclusively inherited disorder. There is likely an inherited predisposition, which leads to the increased likelihood of developing achalasia when exposed to a triggering event [2]. While this theory has good scientific backing on a microscopic level, there has yet to be a comprehensive list of triggering events, which is of obvious clinical importance.

We describe the case of a patient who developed achalasia with a notable history of previous exposure to open-air burn pits during service time with the USAF. A “burn pit” is a man-made hole in the ground where a fire is constructed to dispose of environmental and military waste. Burn pits have been used by the United States Military for decades but gained notoriety in the early 21st century, especially in Iraq and Afghanistan. Joint Base Balad, the largest US military base in Iraq, was quoted to have been burning as much as 147 tons of waste daily [10].

There have been few studies analyzing the health effects of exposure to open-air burn pits, and those completed have conflicting evidence. A study completed in 2010 by the Armed Forces Health Surveillance Center concluded that those who were deployed to bases with known open-air burn pits were not more likely to develop circulatory and respiratory illnesses than service members who were not deployed [11]. However, a 2016 study by Liu et al. found that there were associations between burn pit emission exposure and the incidence of post-deployment self-reported respiratory and cardiovascular conditions [12]. Conflicting results are not unexpected, as the diseases of interest likely have prolonged latency periods, meaning we have yet to see their true incidence in the population exposed.

Air samples obtained from areas surrounding burn pits have been shown to contain carcinogenic compounds such as heavy metals, polychlorinated dibenzo-p-dioxins and dibenzo-p-furans (PCDD/Fs), polycyclic aromatic hydrocarbons (PAHs), and volatile organic compounds (VOCs) [13]. Numerous studies have outlined the negative health effects of exposure to these materials, including enhanced risk of cardiovascular disease secondary to the formation of reactive oxygen species and/or reactive electrophilic metabolites [14], upregulation of systemic levels of pro-inflammatory cytokines [15], and immunocyte degranulation in autoimmune diseases [16]. It is not unreasonable then to suspect that exposure to these chemicals may cause localized inflammation in the gastrointestinal tract, namely, the esophagus, resulting in the development of achalasia in genetically predisposed individuals. More research is required to further analyze this possible link.

## Conclusions

In conclusion, we presented the case of a patient who developed achalasia with a notable history of exposure to open-air burn pits during service time with the USAF. In August of 2022, the US Congress passed the PACT Act, which expanded the healthcare benefits of veterans exposed to burn pits, making the identification of associated conditions a relevant and important endeavor. Our case is the first to suggest a possible link between this exposure and achalasia.
